# Differential contributions of intra‐cellular and inter‐cellular mechanisms to the spatial and temporal architecture of the suprachiasmatic nucleus circadian circuitry in wild‐type, cryptochrome‐null and vasoactive intestinal peptide receptor 2‐null mutant mice

**DOI:** 10.1111/ejn.12631

**Published:** 2014-06-02

**Authors:** S. Pauls, N. C. Foley, D. K. Foley, J. LeSauter, M. H. Hastings, E. S. Maywood, R. Silver

**Affiliations:** ^1^ Department of MathematicsDartmouth College6188 Kemeny HallHanoverNH03755USA; ^2^ Department of NeuroscienceColumbia UniversityNew YorkNYUSA; ^3^ Department of EconomicsNew School for Social ResearchNew YorkNYUSA; ^4^ Department of PsychologyColumbia UniversityNew YorkNYUSA; ^5^ Division of NeurobiologyMRC Laboratory of Molecular BiologyCambridgeUK; ^6^ Department of PsychologyBarnard College of Columbia UniversityNew YorkNYUSA; ^7^ Department of Pathology and Cell BiologyColumbia UniversityNew YorkNYUSA

**Keywords:** circadian rhythm, cluster analysis, cryptochrome, PER2::LUC, spatiotemporal organisation, vasoactive intestinal peptide

## Abstract

To serve as a robust internal circadian clock, the cell‐autonomous molecular and electrophysiological activities of the individual neurons of the mammalian suprachiasmatic nucleus (SCN) are coordinated in time and neuroanatomical space. Although the contributions of the chemical and electrical interconnections between neurons are essential to this circuit‐level orchestration, the features upon which they operate to confer robustness to the ensemble signal are not known. To address this, we applied several methods to deconstruct the interactions between the spatial and temporal organisation of circadian oscillations in organotypic slices from mice with circadian abnormalities. We studied the SCN of mice lacking Cryptochrome genes (Cry1 and Cry2), which are essential for cell‐autonomous oscillation, and the SCN of mice lacking the vasoactive intestinal peptide receptor 2 (VPAC2‐null), which is necessary for circuit‐level integration, in order to map biological mechanisms to the revealed oscillatory features. The SCN of wild‐type mice showed a strong link between the temporal rhythm of the bioluminescence profiles of PER2::LUC and regularly repeated spatially organised oscillation. The Cry‐null SCN had stable spatial organisation but lacked temporal organisation, whereas in VPAC2‐null SCN some specimens exhibited temporal organisation in the absence of spatial organisation. The results indicated that spatial and temporal organisation were separable, that they may have different mechanistic origins (cell‐autonomous vs. interneuronal signaling) and that both were necessary to maintain robust and organised circadian rhythms throughout the SCN. This study therefore provided evidence that the coherent emergent properties of the neuronal circuitry, revealed in the spatially organised clusters, were essential to the pacemaking function of the SCN.

## Introduction

Daily rhythms in physiology and behavior are orchestrated by the suprachiasmatic nucleus (SCN), a bilaterally symmetrical hypothalamic nucleus, which in mammals is comprised of approximately 20 000 neurons. At the level of the individual cell, the key components of the molecular circadian clock entail negative‐ and positive‐feedback loops between clock genes and their protein products (Reppert & Weaver, 2002; Ukai & Ueda, 2010). The genetic analysis of rhythmicity can account for the ability of individual neurons to oscillate in the absence of external input (reviewed in Welsh *et al*., 2010). The elimination of core clock components in individual cells does not necessarily eliminate rhythmicity in the coupled network (reviewed in Welsh *et al*., 2010). The interneuronal connections and the resultant network‐level properties can spare the SCN clockwork from the loss of key signaling elements (Antle *et al*., 2003, 2007; Liu *et al*., 2007; Hu *et al*., 2012). The importance of network properties is also seen in the daily oscillation of circadian gene expression in the SCN, which involves sequential activation of regionally localised clusters of cells, rather than a single simultaneous oscillation of the nucleus as a whole (Quintero *et al*., 2003; Abraham *et al*., 2010; Foley *et al*., 2011; Fukuda *et al*., 2011; Hong *et al*., 2012; Myung *et al*., 2012). Genetic and biochemical evidence suggests a key role for cells in the dorsomedial ‘lip’ of the SCN in initiating the oscillation (Doi *et al*., 2011). Moreover, interference with G protein coupled signaling pathway, specifically Gq‐ (but not Gs or Gi) mediated signaling across the SCN, can reprogram sequential activation (Brancaccio *et al*., 2013). A complete understanding of the SCN as a pacemaker therefore requires a comprehensive analysis of these spatiotemporal sequences, and to achieve that requires the development of tools to first describe the phenomenon and then deconstruct its component features. Finally, such analysis should be applied to genetically modified SCNs to determine the contribution of particular, defined biological mechanisms to these emergent features.

## Relation of cells to networks

Although little is known of its precise network organisation, probably due to the small size of the nucleus and of its individual cells and their fine caliber fibers (van den Pol, 1980; Abrahamson & Moore, 2001; Moore *et al*., 2002; Moore, 2013), it is well established that the SCN is heterogeneous, with individual cells of various sizes, peptidergic phenotypes, and afferent and efferent connections. Furthermore, similar peptidergic cell types are organised in small groups or subregions within the nucleus both spatially (Klein *et al*., 1991; Antle & Silver, 2005) and functionally (Shinohara *et al*., 1995; Noguchi *et al*., 2004; Koinuma *et al*., 2013). A key question is how the activities of individual cells or cell clusters interact to produce the network dynamics of the nucleus as a whole. That the multi‐oscillator system compensates to some extent for the deterioration of individual components is supported by studies of the electrical activity of SCN neurons in aged animals (Aujard *et al*., 2001; Farajnia *et al*., 2014) and of sustained rhythmicity in clock gene mutant animals (Nakamura *et al*., 2002; Liu *et al*., 2007). Physically or chemically dissociating cells from each other results in a decrease in the precision of their circadian oscillation and network interactions that otherwise stabilise their circadian rhythms (Webb *et al*., 2009; Meeker *et al*., 2011). In contrast to dispersed cultures, individual cells within the SCN subregions of intact organotypic slices display great precision in oscillation from cycle to cycle (Evans *et al*., 2011; Foley *et al*., 2011; Fukuda *et al*., 2011).

Circadian oscillation depends on both intracellular and intercellular processes, and studies of animals bearing mutations in clock genes or key SCN peptides or their receptors have contributed to our understanding of the importance of network organisation. The molecular model of the cell‐based oscillator involves interlocked transcriptional/post‐translational negative feedback loops, with rhythmic expression of the transcriptional inhibitors Period and Cryptochrome (Cry), driven by the positive activators Clock and Bmal1. Of specific interest here, Cry‐null mice (lacking both Cry1 and Cry2) are behaviorally arrhythmic immediately on transfer to dim light and brain slices from these animals are also arrhythmic (van der Horst *et al*., 1999; Vitaterna *et al*., 1999). Rhythmicity in individual cells can continue in the absence of Cry‐mediated transcriptional feedback (Maywood *et al*., 2011; Ono *et al*., 2013), indicating that interneuronal signaling is sufficient to maintain circadian pacemaking in the arrhythmic Cry‐null SCN, or that there exist non‐Cry‐based cellular oscillators. Mice lacking vasoactive intestinal peptide (VIP) or its cognate receptor, VIP receptor 2 (VPAC2), are behaviorally arrhythmic, and cellular transcriptional cycles in the SCN are desynchronised and of low amplitude and coherence (Harmar *et al*., 2002; Colwell *et al*., 2003). The effects of a VPAC2 antagonist are even greater than those seen in VPAC2‐null animals, suggesting that other ligands at this receptor may partially substitute for VIP (Brown *et al*., 2007). Consistent with this observation, transcriptional cycles in the VIP‐null and VPAC2‐null SCN can be restored by paracrine cues, including gastrin‐releasing peptide and arginine vasopressin (Harmar *et al*., 2002; Hastings *et al*., 2008; Maywood *et al*., 2011).

## Importance of assessing rhythm stability, amplitude and precision

A problem in evaluating the contribution of cells and the networks in which they participate to circadian rhythmicity is determination of the qualities of the circadian oscillation. Measures of rhythmicity can include indices of amplitude, precision, stability or robustness and spatiotemporal dynamics. Previous studies have classified cells, tissue or animals as either arrhythmic or rhythmic with respect to circadian oscillation, using statistical methods based on curve‐fitting, threshold‐based and physiologically‐based linear differential equations. These methods do not lend themselves to quantitative assessment of the strength of rhythmicity in circadian and other (especially supracircadian) frequencies due to their reliance on the hypothesis that there will be a 24‐h rhythm present. They are also difficult to interpret when the rhythmicity is of low amplitude and/or unstable from cycle to cycle.

## Present goal

The present goal was, first, to examine the spatial and temporal architecture of the multi‐oscillator SCN clock, as revealed by recordings of bioluminescent circadian gene expression in stable, long‐term organotypic slice culture. Second, we define its component features in the wild‐type (WT) SCN and, finally we determine how these features are affected in SCN slices from Cry‐null mutant animals in which the molecular/cellular feedback loop is compromised, and in VPAC2‐null animals that lack VIP‐mediated interneuronal communication. We use quantitative techniques to assess the distinct contribution of both circadian and higher frequencies, and to assess the contribution of the spatial architecture of the SCN to the amplitude and coherence of daily oscillation. We then compare the results of this automated quantitative technique with manual analysis of the same data. To this end, we develop a new technique, i.e. spectral clustering and its associated spectral embedding, building on our previously successful clustering based on the distance between the time series of bioluminescent expression in patches of tissue (Foley *et al*., 2011).

As previously reported, the technique of k‐medoid clustering begins by decomposing the time‐lapse brightness images of the slice data into small regions (‘superpixels’ constituted from square arrays, e.g. in the present case, 2 × 2 pixels). The similarity of the temporal brightness profiles of any pair of these regions is then computed according to a distance function, such as the cosine or correlation distance. Spectral clustering proceeds by envisioning this information as a similarity graph (see Supporting Information for a definition) in which each superpixel of the image is a node, and the similarity of the regions is the weight of the edge connecting them.

The similarity graph interpretation of the data makes it possible to apply powerful graph theoretic techniques, such as optimal graph decomposition through spectral clustering, which provides a solution to what the mathematical literature calls the ‘relaxed N‐cut problem’ [see Shi & Malik (2000) and the Supporting Information]. The relaxed N‐cut problem aims to decompose a weighted graph into constituent parts while doing minimal ‘damage’ to its connectivity. As applied to the SCN data, spectral clustering seeks to decompose the nucleus into constituent parts while retaining the maximal degree of coherent similarity within the parts. Thus, the spectral clusters are spatial regions identified by the similarity graph of the SCN. Spectral clustering, like k‐medoid clustering, can tell us how different tissue regions are organised with respect to their bioluminescence profiles, and provides an independent check on the robustness of the finding that circadian oscillation in the SCN exhibits strong spatial organisation. Spectral clustering also resembles k‐medoid clustering in that neither decomposition provides direct information as to the underlying anatomical and physiological connectivity of the SCN cells. Analysis of spectral and k‐medoid clusters in the SCNs of mutants, however, represents a first step to mapping biological factors governing the anatomy and physiology of the subregions of the SCN and their interactions.

## Materials and methods

### Animals and housing

The WT C57Bl/6, Vipr2^−/−^ (VPAC2‐null) and Cry1^−/−^:Cry2^−/−^ (Cry‐null) mice, bearing a bioluminescent reporter PERIOD2::luciferase transgene (PER2::LUC), were bred at the Medical Research Council (Cambridge, UK) on C57Bl6 backgrounds (Maywood *et al*., 2011). For this study, we used slices from five WT SCNs, five CRY‐null SCNs, and four VPAC2‐null SCNs. Breeding mice were housed under a 12‐h/12‐h light/dim red light cycle with *ad‐libitum* food and water. Mice were killed by cervical dislocation followed by decapitation. No anaesthetic was used. Brains were removed from pups of between 5 and 10 days of age at a time corresponding to the first half of the light phase, and sectioned at 300 μm with a McIlwain tissue chopper. The present studies were obtained in experiments conducted under the Animals (Scientific Procedures) Act 1986 (United Kingdom) and with approval from the ethics committees of the Medical Research Council and the University of Cambridge.

### Suprachiasmatic nucleus slice culture

The slices were left for 1 week to stabilise and during this time they flattened to a thickness of ∼50–100 μm. Bioluminescent emissions from PER2::LUC SCN slices were recorded by charge‐coupled device cameras (Hamamatsu Orca II) as previously described (Maywood *et al*., 2006). In a previous report (Maywood *et al*., 2011), rhythms in bioluminescence were recorded for at least 6 days and typically for 10 days. In that work, waveforms of rhythmic bioluminescence emission from whole slices and individual cells were analysed in brass software [A. Millar (University of Edinburgh, Edinburgh, UK) and M. Straume (University of Virginia, Charlottesville, VA, USA)]. These original recordings were reanalysed to reveal new features using the newly developed procedures described below.

### Visualisation of luciferase in serial frames of the image stacks

In the present study, to visualise changes in bioluminescence over time, raw image stacks were made into individual image sequences using imagej (NIH: http://rsb.info.nih.gov/ij/), and the average brightness of the full frame was taken for each image to create a time series. Peaks and troughs in the time series were computed by taking local maxima and minima over a moving 15‐h window. Using the first trough as time zero, frames at 3‐ or 6‐h intervals were pseudocolored in Volocity (Improvision Inc., Lexington, MA, USA). The rainbow color scale was calibrated to the brightest slice at the top and to extra‐SCN areas at the bottom, making the non‐SCN tissue the same color for each frame.

### Analysis of bioluminescence

#### Image preprocessing

Cosmic rays and other artifacts were removed by setting the highest and lowest 0.0001 quantiles of pixel values in the stack to the mean of their neighborhood (surrounding pixels in the previous, same and next frames) using Mathematica 8 (Wolfram Research, Champaign, IL, USA).

For each of the images from the cleaned luminescence time series, we performed two processing steps. First, we coarsened the image by creating superpixels, i.e. we tiled the image by four pixel (2 × 2) squares and created a coarse image by replacing each of the tiles with a single pixel, the intensity of which was the average of the four pixels of the tile. As the pixels in the image corresponded to a 5 × 5 μm area, the superpixels gave 10 × 10 μm areas. Bilateral SCN images were 128 × 128 superpixels and images of single lobes of the SCN were, consequently, 64 × 128 superpixels. Second, for the spectral clustering analysis, we masked the extra‐SCN area of the image to isolate the tissue from the background.

As previously reported (Foley *et al*., 2011), these steps left the data as stacks of image files with brightness represented by integers. For the cluster analysis, replication of the previously published bilateral analysis can be found in Fig. S2 for each stack. The current analysis compared several methods of analysing each SCN.

#### The k‐medoid cluster analysis algorithm

As previously reported (Foley *et al*., 2011), the analysis of images used an unsupervised learning algorithm, i.e. k‐medoids cluster analysis with the gap statistic (Tibshirani *et al*., 2001), and implemented using Mathematica 8, using the cosine distance function:

$$D_{\cos}\left(u,v\right)=1-\frac{u\cdot v}{\left|u||v\right|}.$$

we chose the cosine distance, in part, to emphasise the similarity of the shapes of the time series, i.e. cosine distance is invariant under rescalings of the data:

$$D_{\cos}\left(\alpha u,\beta v\right)=D_{\cos}\left(u,v\right).$$

This is noteworthy as using cosine distance ensures that differences in basal levels of the time series have no impact on the cluster analysis. Both k‐medoid and spectral clustering (described below) methods place every superpixel into a unique cluster. In the figures containing cluster information, these unique cluster identifications are denoted by distinct colors.

In both the k‐medoid and spectral clustering (described below), we used all available time indices in the recorded series. As a consequence, the durations of the time series were not uniform across specimens. To understand the potential impact of the different durations we also performed clustering over windowed versions of the time series, and found clusters consistent with those revealed in the entire time series (not shown).

#### Quantitative analysis of the clusters

Once the clustering operations were computed, the following analyses were completed on each slice: maps, time series of cluster bioluminescence, and time series of the bioluminescence, as previously reported (Foley *et al*., 2011). Maps indicate the location of each cluster in each slice using a color‐coded spatial map of superpixels, where each color denotes the cluster uncovered in the analysis. The time series of cluster bioluminescence shows the amplitude and phase of each cluster over time. The time series of cluster bioluminescence within each cluster are color‐coded to the spatial map.

As previously noted, light scatters broadly within neural tissue, potentially obscuring the origin of the bioluminescent signal (Foley *et al*., 2011). In the present study, scatter was, however, more difficult to assess, as mutant tissues that do not show coherent spatial or temporal oscillation provide no baseline from which to assess signal leakage into surrounding non‐SCN tissue. Using WT slices from the previous and present studies, we found empirically that restricting analysis to the five or six highest amplitude clusters found using the gap method avoided areas where the intrinsic signal was not dissociable from scatter. Therefore, in the present article, we adopted the convention of showing only the five or six highest amplitude clusters for all analyses. A full comparison to the previous study (Foley *et al*., 2011) can be found in the Supporting Information (Fig. S2).

#### Clustering single‐cell data

Time series corresponding to single cells identified previously (Maywood *et al*., 2011) were clustered using the gap‐method analysis described above. Within each single cell, a 3 × 3 pixel square was identified from which the mean time series was computed. Clusters of cells were compared with superpixel clusters by matching spatial locations.

#### Fourier analysis method

We computed the Fourier spectrum analysis for each cluster of each slice to assess the rhythmicity of the WT and mutant slices. Noise lines were based on the null hypothesis of the uniform Fourier coefficient (white noise), and are shown at 2 SDs from either side of the mean.

#### Frequency filtering method

We filtered the frequency by deleting a notch extending between 18 and 30 h out of the Fourier spectra of each slice, and then computed the reverse transform for time‐series cluster analysis as described above. The notch filter passed all frequencies except those in a defined band. Thus, the amplitude of the notched, filtered Fourier spectra were flat at circadian frequencies.

#### Spectral clustering algorithm

We used spectral clustering (see Chung, 1997; von Luxburg, 2007) to decompose the tissue into regions with coherent behavior of luminescence time series. The algorithm partitioned a similarity network derived from the time‐series data according to the N‐cut optimisation criteria. As with our choice of cosine distance above, we used correlation between time series as our measure of similarity to emphasise the similarity between the shapes of the time series rather than other qualities (rescalings of the time series have no effect on the similarity measures). Details of the algorithm and motivation are provided in the Supporting Information.

#### Spectral embedding

The execution of spectral clustering produced the spectral embedding of the superpixels (a new geometric representation of the superpixels), which is given by mapping the superpixels according to the first *l* eigenvectors of the Laplacian associated with the network. As before, a detailed mathematical description is given in the Supporting Information.

#### Parameter estimation

To execute spectral clustering and construct the spectral embedding we needed to choose three parameters: a scale parameter (*σ*), the number of dimensions in the spectral embedding (*l*), and the number of clusters (*k*). The scale parameter σ indicates which distances (and hence correlations) are deemed important. For example, for σ close to 1, only distances close to 1 (and hence correlations near −1) are mapped to small values in the adjacency matrix. However, using *σ* ≈ 0.3 maps essentially all distances larger than $\sqrt{2}/2$ (hence correlations less than zero) to values very close to zero. In our application, we used *σ* = 0.95 to emphasise the full richness of the correlation structure.

Motivated by two reasons, we selected the number of dimensions in the spectral embedding to be two. First, a two‐dimensional embedding allowed for easy visualisation. Second, scree tests applied to the sequence on non‐zero eigenvalues of the Laplacian (see Supporting Information) showed that every SCN had at least two eigenvalues separated from the bulk of the remaining eigenvalues.

The analyses were carried out using Mathematica 7 and MATLAB 8.2. Code is available by request from the corresponding author.

## Results

### Qualitative and quantitative examination of the suprachiasmatic nucleus in wild‐type, cryptochrome‐null and vasoactive intestinal peptide receptor 2‐null slices

**Figure 1:**
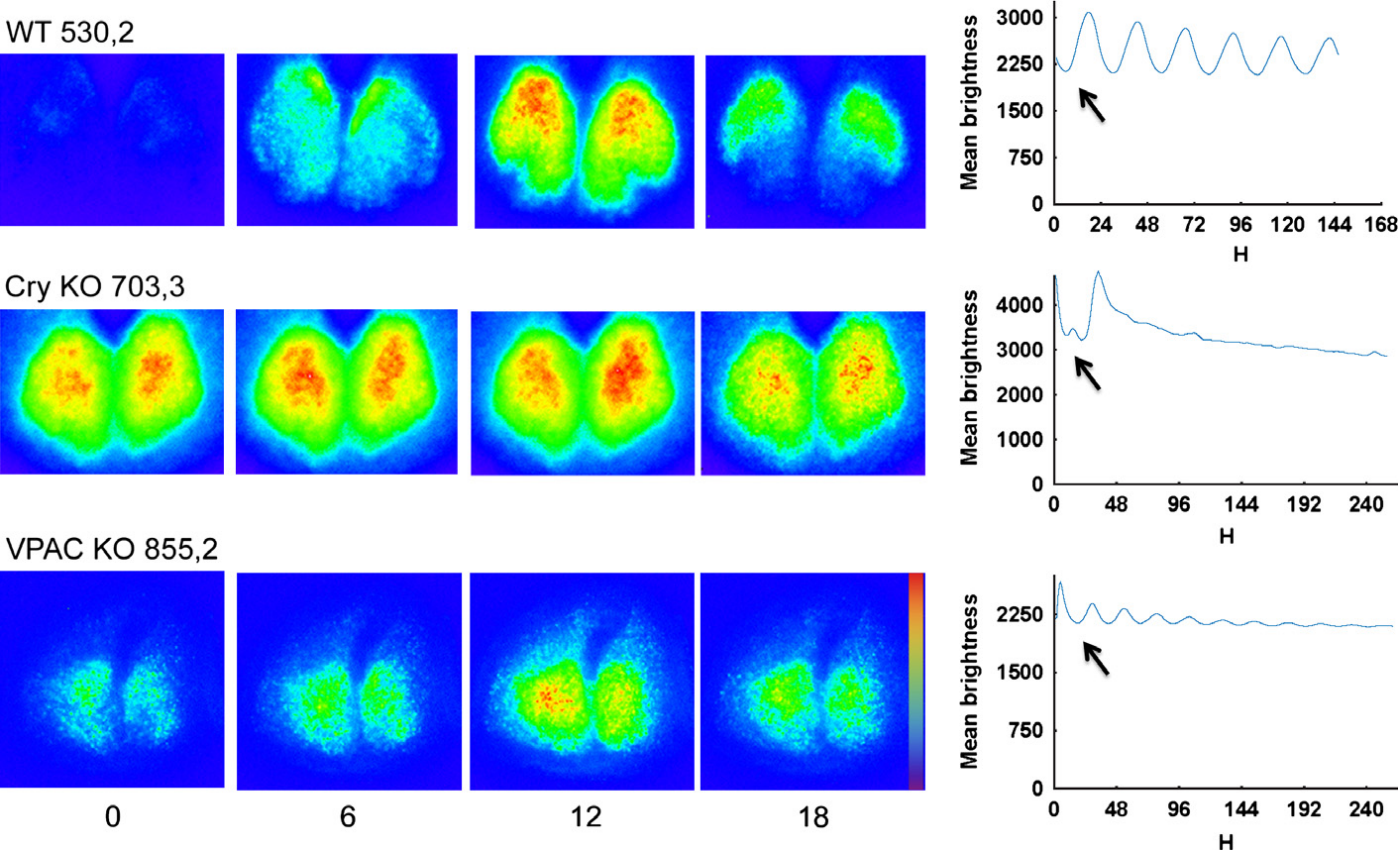
Spatiotemporal pattern in PER2::LUC bioluminescence in SCN for representative slices from WT, Cry‐null and VPAC2‐null mutant animals at 6‐h intervals. Time zero was defined as the trough of the first cycle (indicated by the black arrows). The pseudocolored images are normalised to the brightest slice. Rainbow scale (blue, low and red, high expression) is shown on the righthand side of the last panel. The mean brightness profiles are shown to the right of each row. To facilitate visualisation of differences between WT and mutant slices, the y‐axes are scaled comparably. Due to differences in the length of the brightness profiles, the scales on the *x*‐axis are not uniform.

Changes in the bioluminescence of bilateral WT SCNs are presented in pseudocolored single frames captured at 6‐h intervals throughout the circadian cycle (Fig. 1). Visual inspection of these images shows the phase dispersion of the PER2:LUC signal. Changes in oscillation amplitude over time in WT are similar qualitatively and quantitatively to the previously analysed data [compare present Fig. S1, WT slices with Fig. 4 and present Fig. S2 with Fig. 5 in Foley *et al*. (2011)].

Examination of representative slices from mutant animals, however, shows strikingly different characteristics; whereas the WT SCN shows distinct and stable patterns of expression that progress through the circadian cycle (Fig. 1, top row), the Cry‐null SCN does not oscillate, instead it shows continuous expression in a stable spatial organisation throughout the circadian cycle. Like the Cry‐null slice, the VPAC2‐null SCN also lacks suppression of PER2:LUC expression at any point in the circadian cycle (Fig. 1, second row). Unlike the Cry‐null slice, however, the VPAC2‐null slice does show weak, albeit damped, oscillations in the circadian range (Fig. 1, bottom row). Bioluminescent signals and brightness time series for all slices are shown in Fig. S1.

### Comparison of manual and automated analysis

**Figure 2:**
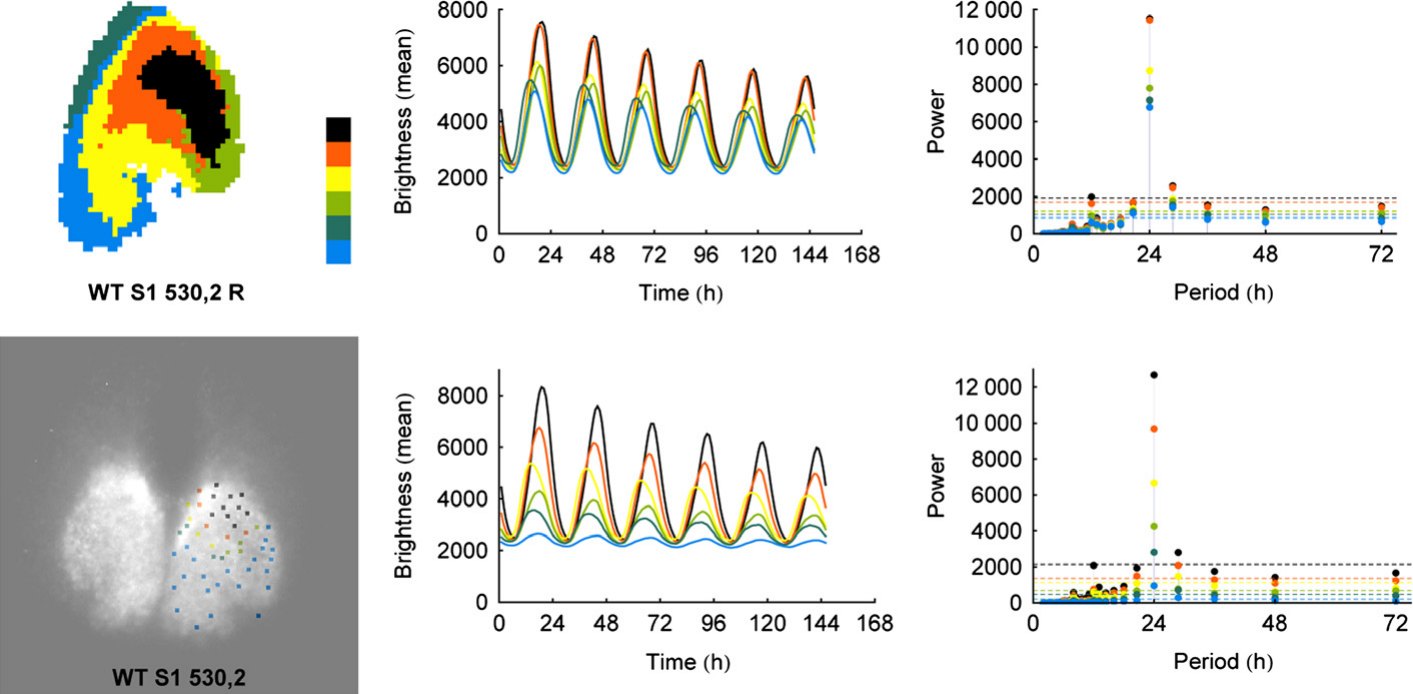
A comparison between the results of the k‐medoid clustering algorithm applied to automatically generated superpixels vs. manually identified cells for a typical WT slice indicates that the results are nearly identical. WT slices are rhythmic and exhibit clearly defined spatial organisation. The left‐most panel shows the color‐coded cluster analysis ‘map’. The color code is shown to the right of the map with the order of cluster coloring from highest (black) to lowest (blue) amplitude. The time series of the mean cluster bioluminescence corresponding to the map is found in the second column. The Fourier analysis is shown on the right. Here and in the following figures, the noise lines are color coded to match the cluster based on the null hypothesis of uniform Fourier coefficient (white noise), and are shown at 2 SDs from either side of the mean.

In Figs 2 and S3 we compare the spatial organisation, mean brightness time series and Fourier spectra, using our automatic superpixel cluster analysis, with results of manually selected cell‐like regions of interest previously reported on these slices (Maywood *et al*., 2011). The top map shows localisation of the top five or six clusters, whereas the bottom map shows clusters of the manually selected cellular regions of interest. The results of the two independent analyses corroborate each other and agree on both spatial organisation and signal strength. This suggests that the developed automatic process corresponds closely with the labor‐intensive manual analysis. In particular, the maps and brightness time series correspond qualitatively (central panels), whereas the manual analysis map highlights the issues with manual analysis such as undersampling of the orange cluster. The Fourier analysis (righthand panels) highlights that all of the five or six highest amplitude clusters show significant circadian power, and at a higher power than is shown in manual analyses of individual cells.

### Cryptochrome‐null slices show stable spatial organisation, but lack circadian rhythmicity

**Figure 3:**
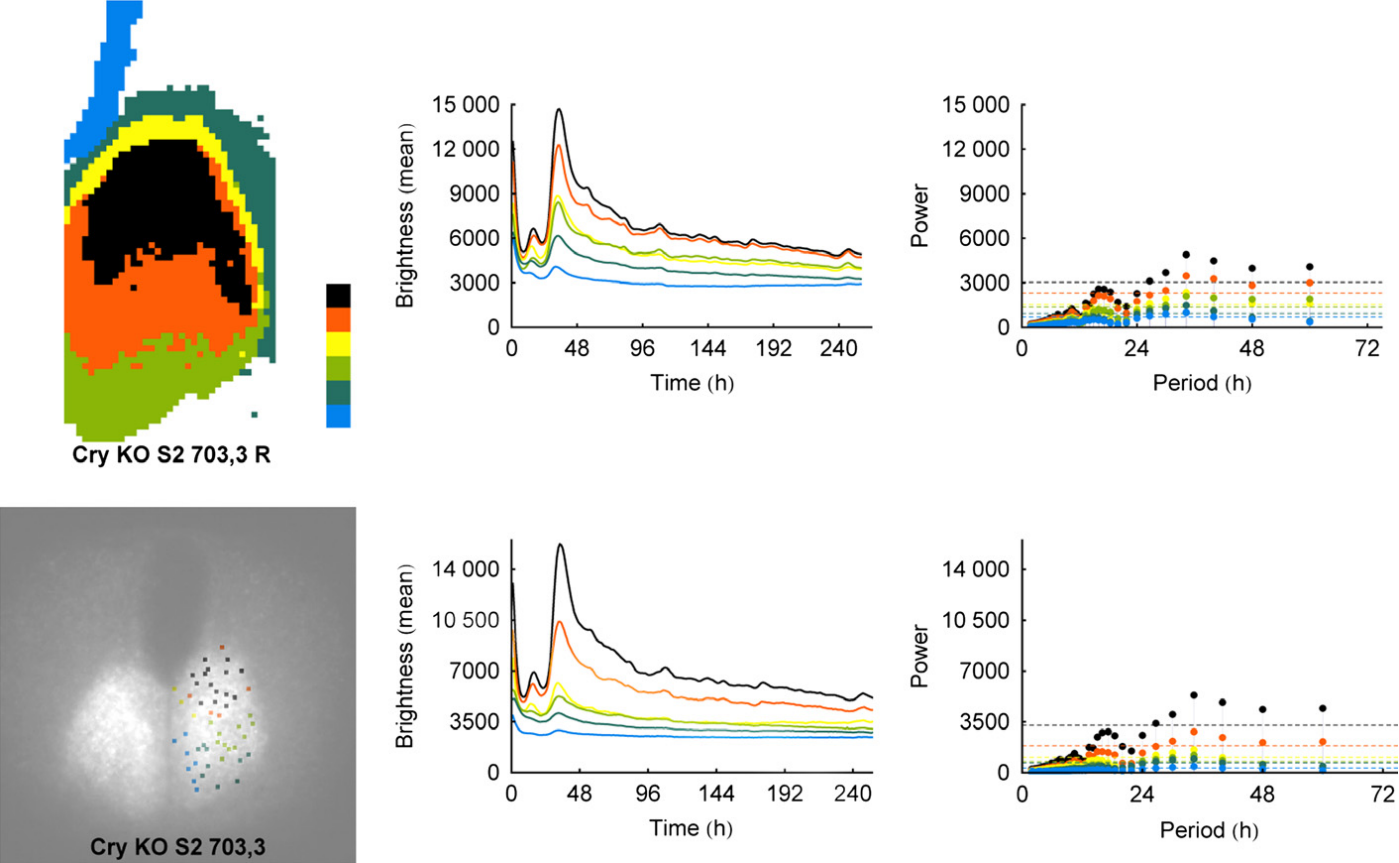
Using the same format as in Fig. 2, we show the results of k‐medoid clustering for a typical Cry‐null slice. As with the WT slices, the results of the analyses for automatically selected regions of interest and manually selected cells are nearly identical. Cry‐null slices are not rhythmic, in agreement with the animal's behavior; surprisingly, however, they have excellent spatial organisation.

Surprisingly, in both the automatic and manual analyses, the representative Cry‐null slice shows a stable spatial organisation (Figs 3 and S4) that is consistent between the analyses. Unsurprisingly and consistent with previous studies (Maywood *et al*., 2011; Ono *et al*., 2013), adult Cry‐null slices are not rhythmic, in agreement with animal behavior. The supracircadian power in Fourier spectra is probably the result of the acute initial stimulation of the tissue upon being introduced into fresh recording medium. Thus, the spatial organisation and temporal organisation are not interdependent and are differentially regulated by Cry proteins.

### Vasoactive intestinal peptide receptor 2‐null slices are heterogeneous

**Figure 4:**
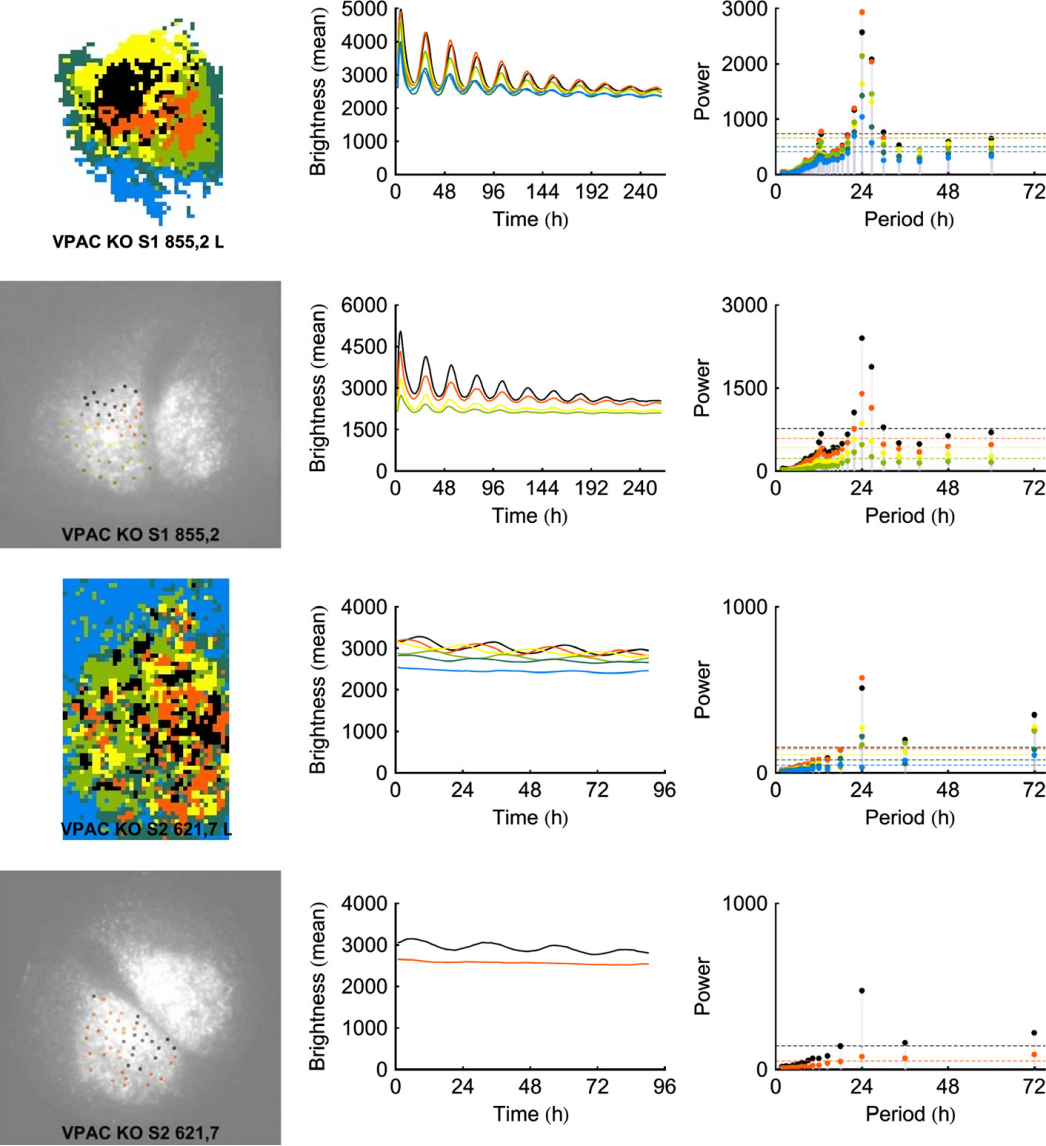
The results of the k‐medoids analysis for VPAC2‐null slices with automatically determined regions of interest (ROIs) show more variability than either WT or Cry‐null slices. The slice bearing spatial organisation has high amplitude rhythmicity while the other lacks both. For each slice, cluster analysis with manually determined ROIs is shown immediately below the automated analysis. Note that, due to differences in the length of the brightness profiles, the scales on the *x*‐axis are not uniform.

The VPAC2‐null mice show variable behavior with about half of the animals showing rhythmic behavior, and half lacking rhythmic behavior (Aton *et al*., 2005; Maywood *et al*., 2006, 2011; Brown *et al*., 2007). Impressively, this variability was reflected in the SCN slices from these mutants. Although all slices showed some power in the circadian range, those with clearer spatial organisation showed higher amplitude oscillation, whereas those lacking coherent spatial organisation had weaker oscillation (Figs 4 and S5 show VPAC2‐null SCN). Comparing automatic and manual analyses, even those slices that lacked clear spatial organisation produced multiple clusters. These clusters were not detected in the manual analyses but were obvious in the automated analyses due to the sensitivity of the clustering algorithm to the size of the data set, again revealing an advantage of automatic analysis.

### The basis of spatial organisation

The foregoing results indicate that both spatial architecture and the individual cellular oscillations contribute to the ability of SCN tissue slices to sustain organised and consistent circadian oscillation *in vitro*. A surprising aspect of the results is that, whereas Cry‐null slices do not show oscillation in the circadian range, they do show robust spatial organisation. To investigate which aspects of the LUC expression underlie the observed spatial organisation detected by the cluster analyses, we used two statistical approaches including notch‐filtering of the Fourier spectra and spectral embedding to investigate a dimensionally reduced signal.

**Figure 5:**
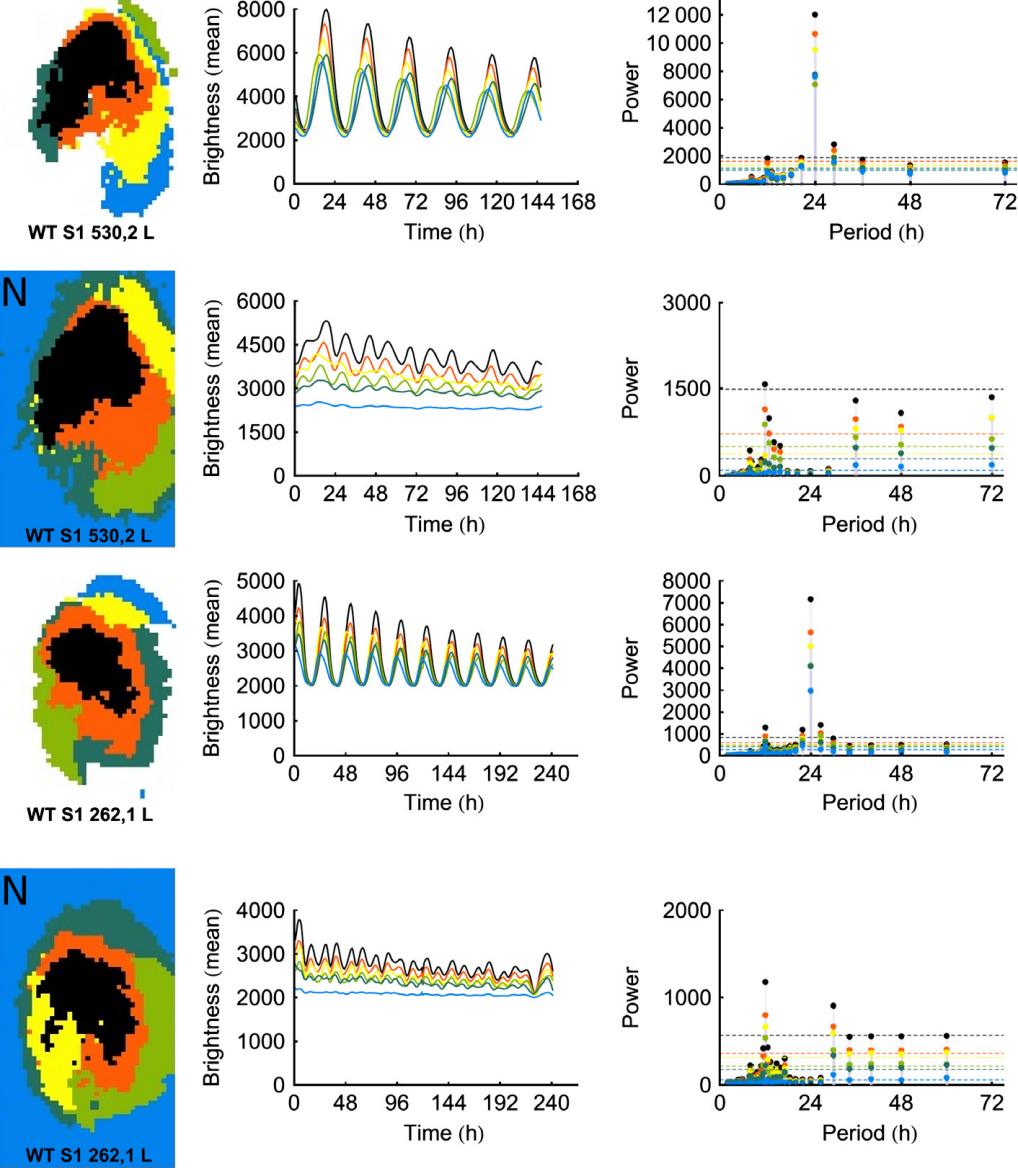
The spatial organisation in WT slices was sustained with non‐circadian frequencies removed by notch‐filtered Fourier spectra between 18 and 30 h. The first and third rows of the figure show the results of the k‐medoids analysis for the original data, whereas the second and fourth rows (marked with an ‘N’) show the results after the signals have been notch‐filtered. Although the spatial organisation is clearly preserved, the notch‐filtered data exhibit a disruption in rhythmicity. Note that, due to differences in the length of the brightness profiles, the scales on the *x*‐axis are not uniform.

Our preliminary hypothesis was that spatial organisation required frequencies outside the circadian range. The cluster map, time series and Fourier analysis of the left SCN for two WT slices are shown in the first and third rows of Fig. 5 (additional slices in Fig. S6). To test whether the spatial organisation was sustained with only non‐circadian frequencies, we notch‐filtered the Fourier spectra between 18 and 30 h, and recomputed the cluster analyses, as can be seen on the second and fourth rows of Fig. 5 (marked with an ‘N’). Clustering in WT slices remained remarkably stable with the circadian component of the oscillation removed. Inspection of the notch‐filtered time series and Fourier power spectra suggests that this is primarily due to ultradian oscillations, as each slice presents significant Fourier power in the ultradian range, whereas not all slices present significant Fourier power in the infradian range.

### Elucidating variable spatiotemporal organisation using spectral embedding

**Figure 6:**
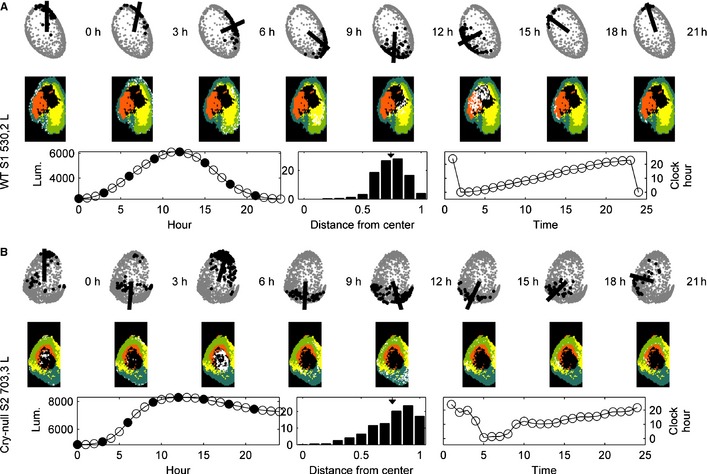
The ability of spectral embedding to demonstrate the link between spatial and temporal organisation of the SCN. The two rows (labeled A and B) depict different slices, each of which is analysed separately for the left (shown here) and right (shown in Fig. S7) nucleus. In each subfigure, there are three rows. Images in the top two rows show sequential changes in time. In the top row of ‘clock faces’ given by the spectral embedding, the black circles are associated with spatial locations with the highest response at that time, whereas the line indicates the mean direction of all of the black circles. In the second row, the colored regions are the clusters identified by spectral clustering, with the white dots indicating the spatial locations associated with the black circles in the top row. The three figures on the bottom row are, from left to right, the mean signal over the period of observation, a histogram of the distances of the spectrally embedded superpixels from the center of the embedding (arrowhead indicates the mean), and the phase plot over the entire time series. (A) WT slices show a strong connection between spatial and temporal organisation. The histogram indicates a well‐delineated circular spectral embedding. (B) In Cry‐null slices, spectral clustering echoes the earlier clustering results, showing clear spatial organisation. However, in contrast to the WT slices, the spectral embedding provides no link from the spatial organisation to temporal organisation, indicating that the tissue does not exhibit such organisation. The histogram indicates less well‐delineated circles than in the WT slice.

We used the spectral embedding to investigate the link between the temporal and spatial organisation of WT and mutant SCN. In WT animals, the spectral embedding of the superpixels provides a direct link between coherent spatial organisation and coherent temporal organisation (as the mean luminescence profile moves through a complete circadian rhythm, the spectral embedding identifies sequential spatially coherent regions whose local profiles peak at that time). To see this precisely, we focus on the circle formed by the spectral embedding (see Fig. 6A). The temporal organisation is encoded by the position of the superpixels on the circle itself. First, the superpixels that are close in the spectral embedding have time series that reach maxima at similar times. Second, moving clockwise around the circle corresponds to moving this peak forward through the 24‐h period. Because of this, it is natural to interpret the spectral embeddings as 24‐h ‘clock faces.’ The distribution on the clock face is associated with proximity in the spectral embedding, and clusters of superpixels in the spectral embedding correspond to spatially contiguous areas of tissue in the SCN. (Thus, if the clusters have no organisation, the points would be spread throughout the clock face, whereas in perfectly organised clusters, the points would be perfectly aligned on the periphery of the clock face.) The results show that the combination of these two aspects (proximity in the spectral embedding, and clusters of superpixels) connects contiguous spatial structures with the temporal structure of the signal; a clock face with a well‐delineated circle (with many pixels on the periphery and few pixels in the interior of the disk) indicates a stronger spatial–temporal organisation. Conversely, a clock face with many pixels in the interior indicates a less robust connection between spatial and temporal properties. It is noteworthy that there is no methodological reason to predict this extremely interesting result.

As indicated by the example in Fig. 6A, WT slices (top three rows) exhibit linked spatial and temporal organisation over the mean cycle of the circadian period. The top row shows the spectral embedding of the representative WT slice. The line within the ‘clock face’ in each figure is the average direction of the superpixels with peak luminescence at the time indicated above the image. The superpixels used to create the average are denoted by black‐filled circles in the spectral embedding. The middle row shows images of the SCN tissue with the same superpixels marked in white. The colors of the other tissue superpixels are given by the clusters found through spectral clustering (*σ* = 0.95, *l* = 2, *k* = 5). The bottom row of each panel shows the time series in the lower left corner as the mean time series overall superpixels, where the filled circles correspond to the snapshots shown in the top two rows. The middle panel of the bottom row is a histogram of distances of the spectrally embedded superpixels to the center of mass of the entire spectral embedding divided by half the diameter of the embedding. The histogram thus indicates the extent to which the spectral embedding forms a well‐delineated circle and, hence, the strength of the spatiotemporal coupling. The mean of these distances is indicated by the inverted arrowhead at 0.74. In this figure, the fact that the bulk of the histogram is grouped towards the righthand side indicates a clean circular embedding given by the spectral coordinates. The righthand plot of the third row plots the ‘clock‐hand angle’ vs. time (in hour). This plot gives the average direction shown in the top row, but for all time points. As can be seen in the WT slices, strong circadian organisation is reflected by an orderly progression of peak expression from the top of the clock (0 h) clockwise through 24 h to return to the top of the clock. Further evidence of these conclusions is demonstrated in the Supporting Information figures. Figure S7 shows the right SCN in each of the representative slices for which Fig. 6 shows the left side. Additional WT SCNs are shown in Fig. S8 (left lobe) and Fig. S9 (right lobe).

### Cryptochrome‐null slices exhibit spatial organisation without temporal organisation

The Cry‐null (Fig. 6B) specimens provide a first contrast to the results of the spectral embedding of the WT animals. Echoing the earlier clustering results, we see a distinct spatial organisation given, as with the WT animals, by clusters in the spectral embedding. However, these clusters are no longer linked to smoothly increasing peaks in their luminescence profiles. Although the superpixels that cluster in the spectral embedding are again grouped in spatially contiguous regions, they do not move around the ‘clock face’ of the spectral embedding in the same orderly way. In this specimen, the sequence begins moving clockwise but eventually bounces back and forth irregularly. This is further indicated in the clock face itself (in contrast to the WT specimens, the spectral embeddings are not well‐delineated circles, having much less defined edges and interior). This is confirmed by the distance histogram, which shows a much broader distribution with mean at 0.77. The movie, SM2, illustrates the disconnect between the spatial and temporal structures as we see the ‘clock hand’ progressing around the face until it becomes stuck at the same phase in sequential cycles. Figures S10 (left SCN) and S11 (right SCN) show additional analyses of Cry‐null SCN supporting these findings.

### Vasoactive intestinal peptide receptor 2‐null slices can exhibit temporal organisation without spatial organisation, but the phenotype of these mutants is much less consistent

**Figure 7:**
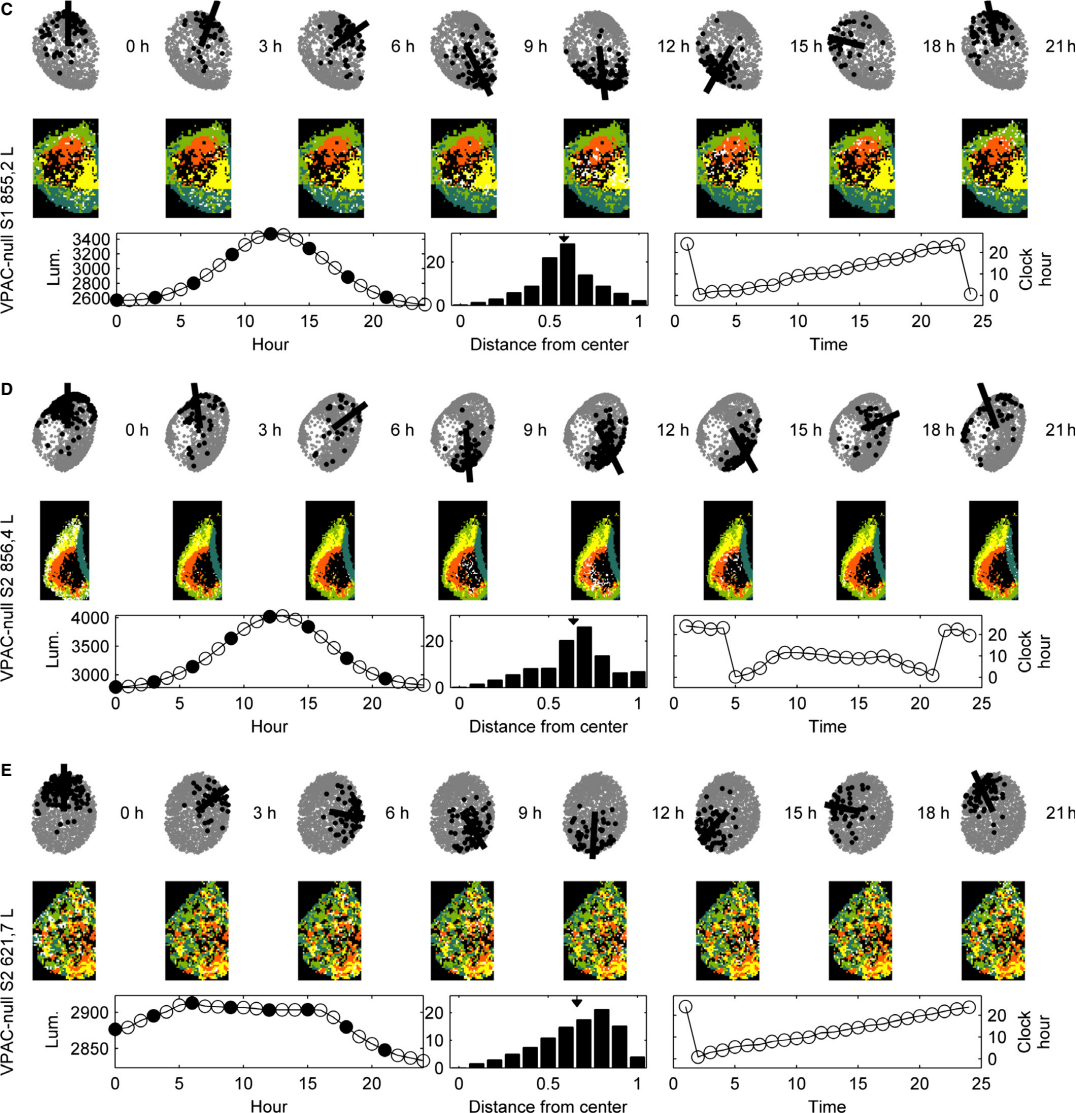
This figure repeats the analyses shown in Fig. 6 for three VPAC2‐null left SCNs (the right lobes are shown in Fig. S7). (C) In this VPAC2‐null slice, spectral embedding connects the temporal organisation to an albeit weaker spatial organisation. (D) This VPAC2‐null slice exhibits both spatial and temporal organisation, but the spectral embedding does not link the two. (E) This VPAC2‐null slice shows temporal organisation without spatial organisation.

Unlike the WT or Cry‐null mutants, there is no generic observation concerning the VPAC2‐null mutants using this technique. We expect that the differences in these specimens arise due to differences among VPAC2‐null animals, as previously reported at the behavioral level of analysis (Aton *et al*., 2005). For sample 855,2 L, the spectral embedding connects temporal organisation with spatial organisation just as in the WT specimens, albeit with spatial organisation that is less clearly defined (Fig. 7C). In contrast, slice 856,4 L (Fig. 7D) exhibits both spatial and temporal organisation, but the spectral embedding does not connect the two as it does in the WT specimens.

The remaining VPAC2‐null mutants do not exhibit spatial organisation and have varying degrees of temporal organisation (Fig. 7). For example, in one case (Fig. 7E), we see clear temporal organisation in the signal that is reflected in the clock face given by the spectral embedding, but linked to spatial areas that are neither organised nor contiguous. In all cases, the circles formed in the spectral embeddings are much less well defined than in the WT specimens, as demonstrated by the histograms in the figures. Moreover, the distributions are much more spread out.

The histograms in each case reflect that the spectral embeddings are less coherently structured, still roughly circular but they are filled disks rather than circles. The means for the three representative samples shown are 0.58, 0.64 and 0.66, respectively (Fig. 7C–E for the left SCN). The VPAC2‐null specimens for the right SCN are shown in Fig. S7C–E and an additional VPAC2‐null specimen is shown in the bottom row of Fig. S10 (left SCN) and Fig. S11 (right SCN).

## Discussion

In our previous studies we showed that, in WT SCNs, the orderly spatial architecture and the amplitude of circadian oscillation are correlated, implying some common regulation. The surprising result in the present analysis is the discovery that the occurrence of an orderly spatial architecture of the SCN is independent of the existence of circadian oscillation i.e. temporal and spatial coding in the SCN are dissociable. Specifically, spatially coherent Cry‐null slices do not express circadian oscillations, whereas some spatially incoherent VPAC2‐null slices do oscillate. Thus, spatial and temporal coding in the circadian circuit of the SCN are independently sensitive to disruption of intracellular and intercellular mechanisms. Further analysis of this stereotypical behavior of the SCN may inform how behaviorally relevant, emergent properties arise from relatively simple circuits in the mammalian brain.

### Automated cluster analyses are much more efficient than manual analyses and provide equal or better quality of results

With the development of more sensitive fluorescent and bioluminescent reporters and sophisticated imaging equipment, it has become possible to interrogate the spatiotemporal architecture of the circadian cycles of gene expression in the SCN. Imaging data have therefore become increasingly important in our understanding of functional organisation of the SCN, serving as a prototypical example for neural circuitry. Previous analyses have relied on manual cell region of interest specification or averaging across multiple slices to provide substantial insights (Yamaguchi *et al*., 2003; Evans *et al*., 2011; Enoki *et al*., 2012; Maywood *et al*., 2013). These are time‐intensive, subject to unconscious experimenter selection biases and ignore possible artifacts caused by scatter of light and/or small movements of cultured tissue and cells. In addition, averaging across animals can mask individual slice variability, possibly as a function of tissue selection. More recently, automated approaches with less selection bias and greater capacity have been developed to characterise circuit‐level patterns of gene activity. These have included computation of the center of mass of bioluminescence and its migration over circadian time (Brancaccio *et al*., 2013) as well as more comprehensive hierarchical clustering to define phase groups and their responses to prior daylength or pharmacological addition of synchronising cues (Myung *et al*., 2012; Evans *et al*., 2013).

The methods applied in the current study offer further opportunities for formal analysis. They are both robust and automated, providing rapid analysis on the single slice level, suitable for interslice comparison but avoiding potential selection bias and reducing signal contamination caused by the scatter of light in the tissue. Importantly, the experimental findings presented here are based on consistent results from two different unsupervised cluster‐learning algorithms that make no assumptions about tissue‐level organisation: spectral embedding and cosine distance k‐medoids. In addition, the work provides algorithms that make a substantial methodological contribution in demonstrating the efficacy of automated analyses of bioluminescent imaging, as compared with manually selected sparse samples of cell‐like regions of interest. Finally, a reassuring point of the results is that WT slices show consistent spatiotemporal LUC::PER2 expression despite differences in tissue preparation protocols used [e.g. slice thickness, media, tissue age, in Foley *et al*. (2011) and Maywood *et al*. (2011)], i.e. the automated analyses give similar results, but with greater refinement, require less effort and allow for deeper interrogation of the observed patterns.

### Spatial organisation without circadian oscillation

A surprising result in the present study is the robust spatial architecture of the SCN of Cry‐null animals in the absence of circadian rhythms. Such findings indicate that oscillations at the circadian time scale are not necessary to spatially organise the SCN tissue. In these mutants, behavioral rhythmicity is absent immediately when mice are placed in the dark. That the infrastructure for producing oscillations is present is indicated by the fact that neonatal Cry‐null SCNs exhibit coherent oscillation for a period of up to 10 days after birth, whereupon it is lost (Ono *et al*., 2013).

With this background of circadian incompetence in the adult Cry‐null slice, it is possible that spatial organisation of the SCN arises through the significant Fourier power spectra in the ultradian range. This is consistent with the present results showing strong spatial coherence in notch‐filtered (18–30 h) WT slices (Fig. 5). Calcium imaging data demonstrate similar tissue organisation at shorter time scales, demonstrated by the synchronised phasic perturbations found in Fig. 1C in Enoki *et al*. (2012). That the SCN of arrhythmic Cry‐null animals is fundamentally capable of expressing (imposed) circadian oscillation is seen in co‐culture work where paracrine signals from a WT slice can restore rhythmicity (Maywood *et al*., 2011). Furthermore, the rhythm in Cry‐null is rapidly restored (Maywood *et al*., 2011). Importantly, this indicates that the network organisation, seen in the spatial architecture revealed here, is in place and readily responds to appropriate signals.

The spectral embedding analysis supports the results of cluster analysis and, in WT, demonstrates the regular spatial procession in peak bioluminescence in repeated cycles (movie SM1). Unlike WT slices, however, in Cry‐null SCNs further activity does not proceed from cluster to cluster in an organised fashion, as indicated by the sharp ‘swings’ of the clock face back and forth at the end of a smooth movement. Thus, even though local spatial structure is retained, large‐scale spatial structure is not.

### Circadian oscillation without spatial organisation

A notable feature of the VPAC2‐null slices is the interslice variability. At one extreme, these slices almost resemble the WTs in spatial architecture and oscillation (although their oscillations do eventually damp), whereas at the other extreme, they have no discernible organisation of either feature. Critically, this variability is also seen in the behavior of VPAC2‐null animals (Hughes & Piggins, 2008). Although the mechanism for this is not clear, it is possible that, in some slices, gastrin‐releasing peptide or arginine vasopressin is sufficient to produce rhythmicity in the absence of VIP signaling (Brown *et al*., 2005; Maywood *et al*., 2011), or that unknown epigenetic effects contribute.

An unanswered question is what mechanism underlies the transition from one cluster to the next. It is not known whether this feature is derived from cellular oscillation, as the result of network properties, or an interaction between them. It remains to be determined whether the paracrine signaling peptides that restore rhythms act to enable orderly progression of oscillation of distinct clusters, i.e. the sequence of activation of clusters is carried by a peptidergic temporal code. A similar code has been proposed to operate in the context of SCN outputs, insofar as the overlapping, rhythmic release of multiple SCN locomotor factors establishes sharp boundaries that trigger time‐stamped events such as activity onset (Kraves & Weitz, 2006).

### Function of spatial organisation

Extending this view of an internal temporal code, the sequential activation across the SCN circuit may be a mechanism whereby particular neurochemically or neuroanatomically specific populations of SCN efferent pathways are activated, thereby establishing a suitably coordinated sequence of activation and inhibition of targets, and their dependent behaviors and physiology.

In the present study, we find that both tissue‐level organisation and cellular oscillation are required to produce a stable and robust circadian rhythm. Although spatial architecture of the SCN has not been taken into account in previous studies of clock gene mutations, there has been attention to its function in the context of photoperiodism. Here, tissue‐level SCN architecture provides a temporal basis for encoding of seasonal variation of daylength (Inagaki *et al*., 2007; Meijer *et al*., 2007; Sosniyenko *et al*., 2009).

The SCN is generally considered unique in that the time scale of 24 h is not explicable in terms of the time course of well understood phenomena such as electrical activity, neurotransmitter release, and gene transcription and translation. Our results indicate novel aspects of the structure of the SCN, which are probably related to the complex dynamics that govern a robust 24‐h cycle. The necessity of tightly linked temporal and spatial structures for the existence of a high‐amplitude robust circadian rhythm indicates functional spatially contiguous units within the SCN that play a central role at specific points in the cycle. The link between spatial organisation and non‐circadian signals points towards heterogeneity in the oscillation, which we hypothesise contributes to the robustness of the overall signal as well as helping to facilitate the change in time scale.

## Supplementary Material

Fig. S1. Spatiotemporal patterns in PER2::LUC bioluminescence in SCN for all slices, analysed in 3 h intervals. Time zero was defined by the trough of the first cycle (indicated by black arrows), and the pseudocolored images are normalised to the brightest slice. The mean brightness time series for bioluminescence for each slice is shown to the right of each row. As noted in text, the analyses performed in this paper assess the amplitude of oscillation and are not sensitive to the baseline brightness of the image. The figures retain the same axes for all slices, to facilitate comparison among WT and mutant tissues shown on subsequent figures. The color scale is shown on the right side of the last panel.
Fig. S2. For each WT slice, we show (from left to right) the “map” showing the top six clusters produced by the k‐medoids algorithm, and the mean brightness time series for each clusters, following previous work (Foley *et al*., 2011). In addition, we provide the Fourier power spectrum for each cluster.
Fig. S3. The results of the k‐medoid analysis for all WT samples are shown for the automatically determined ROIs. In the first three columns, from left to right, we show the “map” of the top six clusters in the tissue, the mean signals over the clusters, and the Fourier power spectra. The right hand panels show the same analyses for manually identified cells.
Fig. S4. Similarly to Figure S3, we show the results of the k‐medoid analysis for all Cry‐null samples with automatically determined ROIs. In the first three columns, from left to right, we show the top six clusters in the tissue, the mean signals over the clusters, and the Fourier power spectra. The right hand panels show the same analyses for manually identified cells.
Fig. S5. The results of manual and automatic cluster analysis are shown for two additional VPAC2‐null SCN.
Fig. S6. As in Figure 5 of the main text, the first, third, and fifth rows of the figure show the results of the k‐medoids analysis for the original data while the second, fourth, and sixth rows (marked with an N) show the results after the signals have been notch filtered. As with the examples in Figure 5, while the spatial organisation is clearly preserved, the notch filtered data exhibits a disruption in rhythmicity.
Fig. S7. We show the same analyses as in Figures 6 and 7 for the right lobes of the SCN in that Figure.
Fig. S8. We show the results of following the procedure used to create Figures 6 and 7 for all additional WT animals (left lobes).
Fig. S9.We show the results of following the procedure used to create Figures 6 and 7 for all additional WT animals (right lobes).
Fig. S10. We show the results of following the procedure used to create Figures 6 and 7 for the left lobes of all additional Cry‐null SCN (top rows) and one VPAC2‐null SCN (last row).
Fig. S11. We show the results of following the procedure used to create Figures 6 and 7 for the right lobes of all additional Cry‐null SCN (top rows) and one VPAC2‐null SCN (last row).

Video S1. The video shows an animation of the right SCN of the WT specimen shown in Fig. 6A and Fig. S7A.

Video S2. The video shows an animation of the right SCN of the WT specimen shown in Fig. 6B and Fig. S7B.
