# Supplementary material for: Differential contributions of intra‐cellular and inter‐cellular mechanisms to the spatial and temporal architecture of the suprachiasmatic nucleus circadian circuitry in wild‐type, cryptochrome‐null and vasoactive intestinal peptide receptor 2‐null mutant mice
Source: Eur J Neurosci. 2014 Jun 2;40(3):2528–40. doi: 10.1111/ejn.12631 (PMC4159586; doi:10.1111/ejn.12631)
Supplement: Supplementary file 1 — Fig. S1. Spatiotemporal patterns in PER2::LUC bioluminescence in SCN for all slices, analysed in 3 h intervals. Time zero was defined by the trough of the first cycle (indicated by black arrows), and the pseudocolored images are normalised to the brightest slice. The mean brightness time series for bioluminescence for each slice is shown to the right of each row. As noted in text, the analyses performed in this paper assess the amplitude of oscillation and are not sensitive to the baseline brightness of the image. The figures retain the same axes for all slices, to facilitate comparison among WT and mutant tissues shown on subsequent figures. The color scale is shown on the right side of the last panel. Fig. S2. For each WT slice, we show (from left to right) the “map” showing the top six clusters produced by the k‐medoids algorithm, and the mean brightness time series for each clusters, following previous work (Foley et al., 2011). In addition, we provide the Fourier power spectrum for each cluster. Fig. S3. The results of the k‐medoid analysis for all WT samples are shown for the automatically determined ROIs. In the first three columns, from left to right, we show the “map” of the top six clusters in the tissue, the mean signals over the clusters, and the Fourier power spectra. The right hand panels show the same analyses for manually identified cells. Fig. S4. Similarly to Figure S3, we show the results of the k‐medoid analysis for all Cry‐null samples with automatically determined ROIs. In the first three columns, from left to right, we show the top six clusters in the tissue, the mean signals over the clusters, and the Fourier power spectra. The right hand panels show the same analyses for manually identified cells. Fig. S5. The results of manual and automatic cluster analysis are shown for two additional VPAC2‐null SCN. Fig. S6. As in Figure 5 of the main text, the first, third, and fifth rows of the figure show the results of the k‐medoids analysis for the o [file ejn-40-2528-s9.pdf]

Supplementary figures:

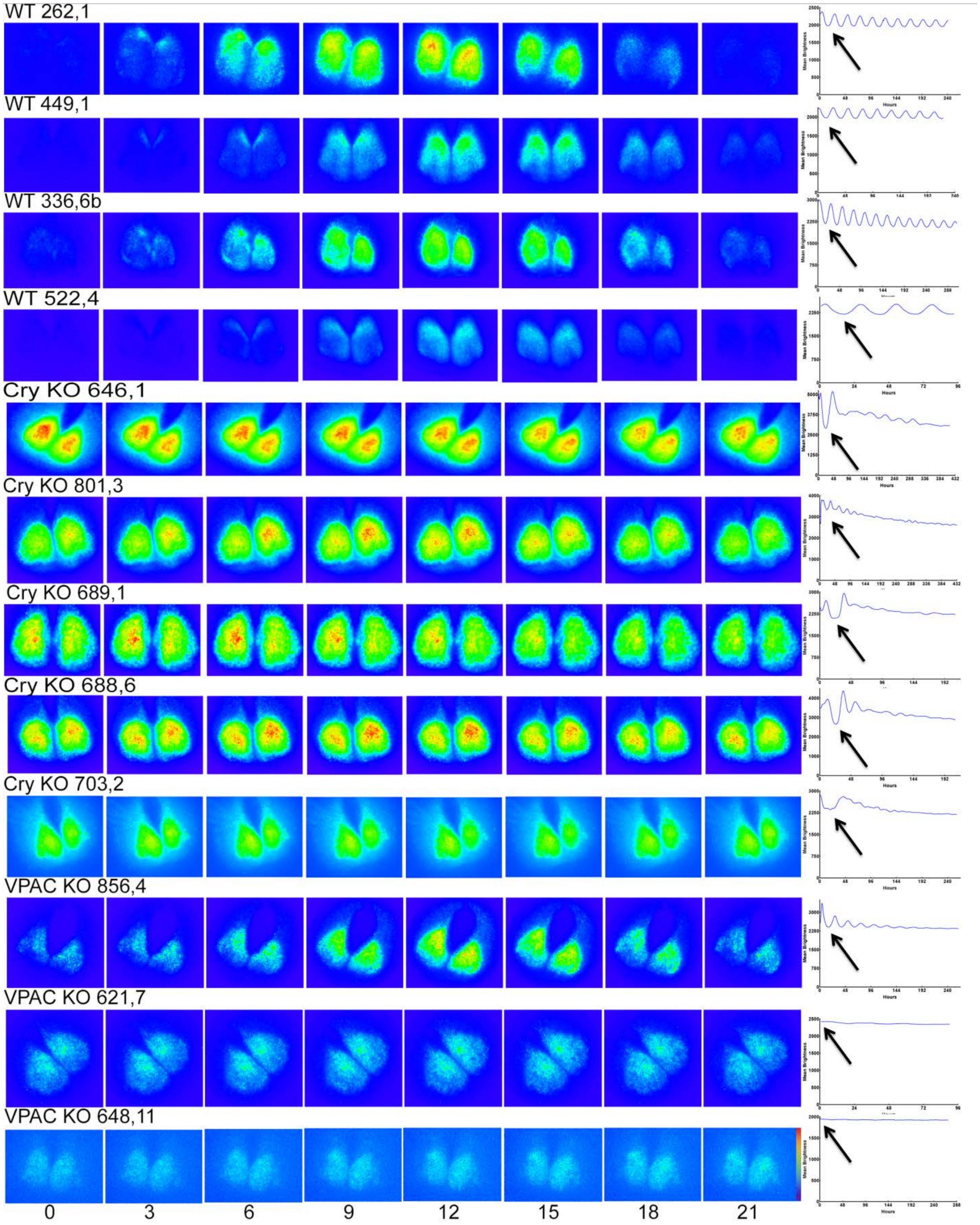

Figure S1: Spatiotemporal patterns in PER2::LUC bioluminescence in SCN for all slices, analyzed in three hour intervals. Time zero was defined by the trough of the first cycle (indicated by black arrows), and the pseudocolored images are normalized to the brightest slice.

The mean brightness time series for bioluminescence for each slice is shown to the right of each row. As noted in text, the analyses performed in this paper assess the amplitude of oscillation and are not sensitive to the baseline brightness of the image. While the y-axes have been uniformly scaled, due to the different lengths of the time series, the x-axes have different scales. The color scale is shown on the right side of the last panel.

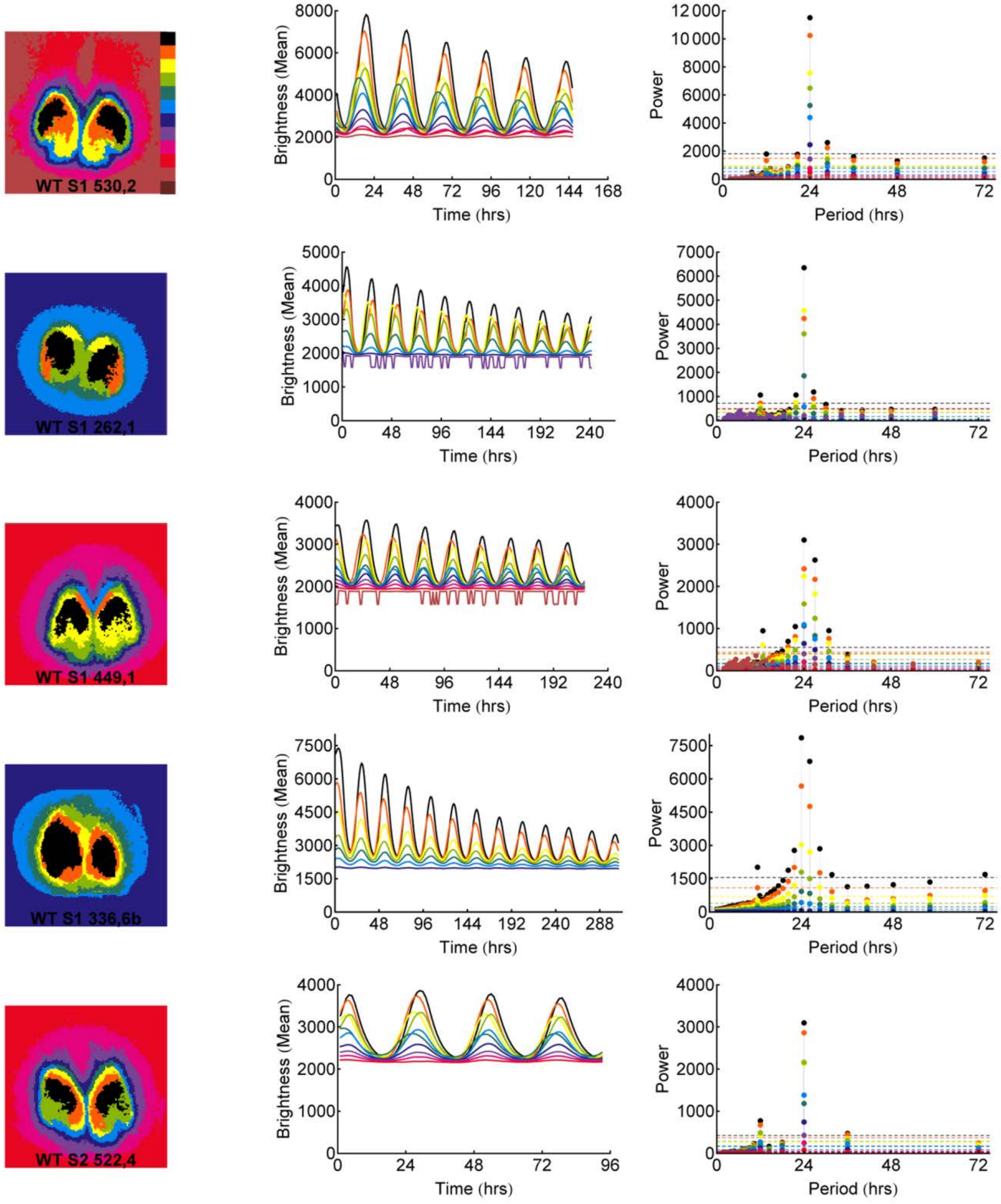

Figure S2: For each WT slice, we show (from left to right) the “map” showing the top six clusters produced by the k-medoids algorithm, and the mean brightness time series for each

clusters, following previous work (Foley et al., 2011). In addition, we provide the Fourier power spectrum for each cluster. The color scale is shown on the right side of the first “map”. Note that, due to the different lengths of the time series, the x-axes have different scales.

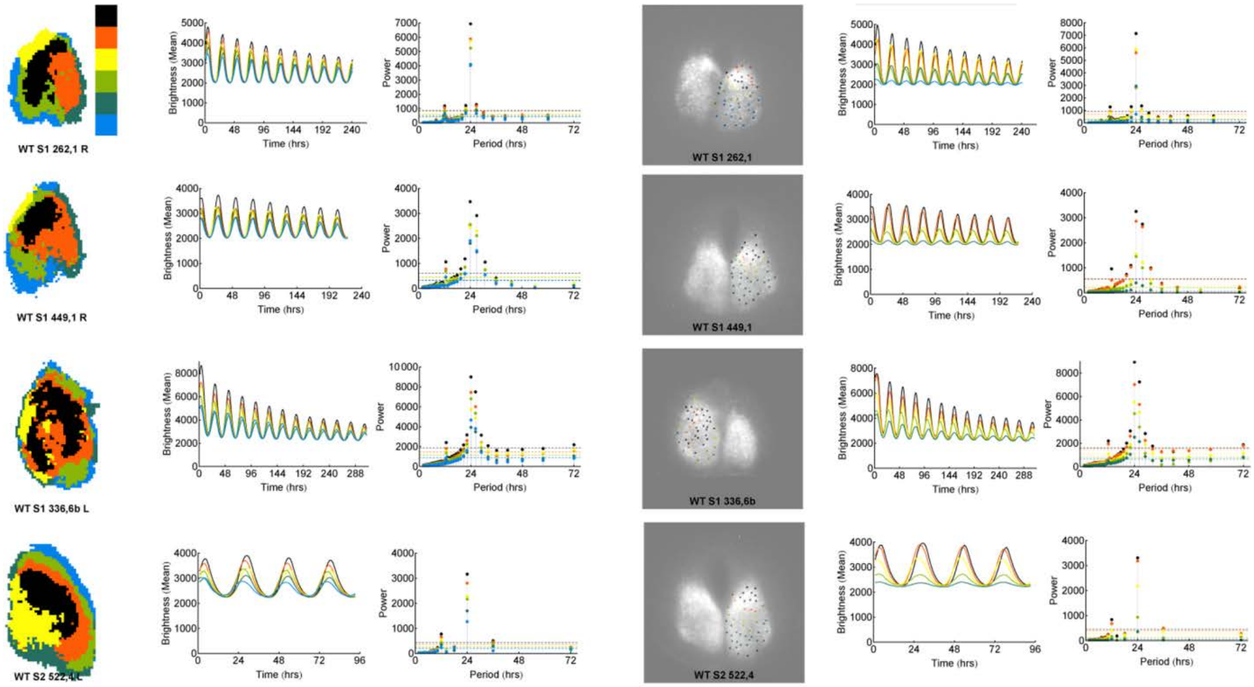

Figure S3: The results of the k-medoid analysis for all WT samples are shown for the automatically determined ROIs. In the first three columns, from left to right, we show the “map” of the top six clusters in the tissue, the mean signals over the clusters, and the Fourier power spectra. The right hand panels show the same analyses for manually identified cells. Note that, due to the different lengths of the time series, the x-axes have different scales.

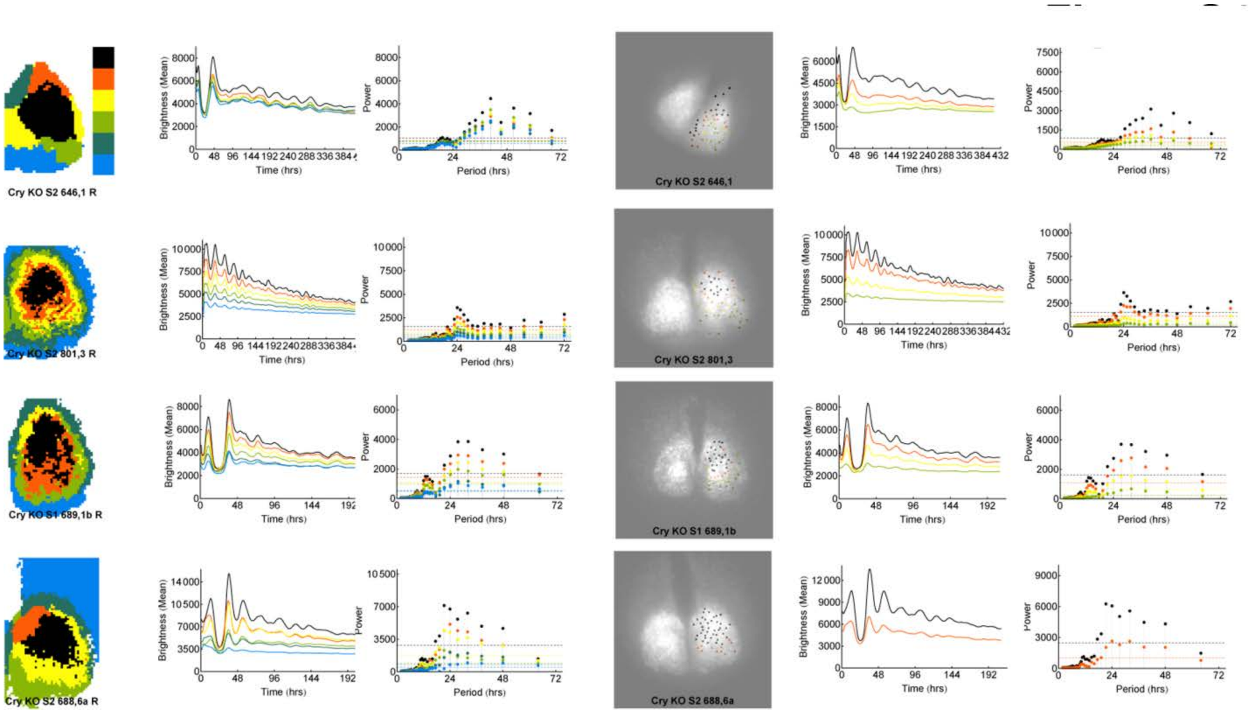

Figure S4: Similarly to Figure S3, we show the results of the k-medoid analysis for all Cry-null samples with automatically determined ROIs. In the first three columns, from left to right, we show the top six clusters in the tissue, the mean signals over the clusters, and the Fourier power spectra. The right hand panels show the same analyses for manually identified cells. Note that, due to the different lengths of the time series, the x-axes have different scales.

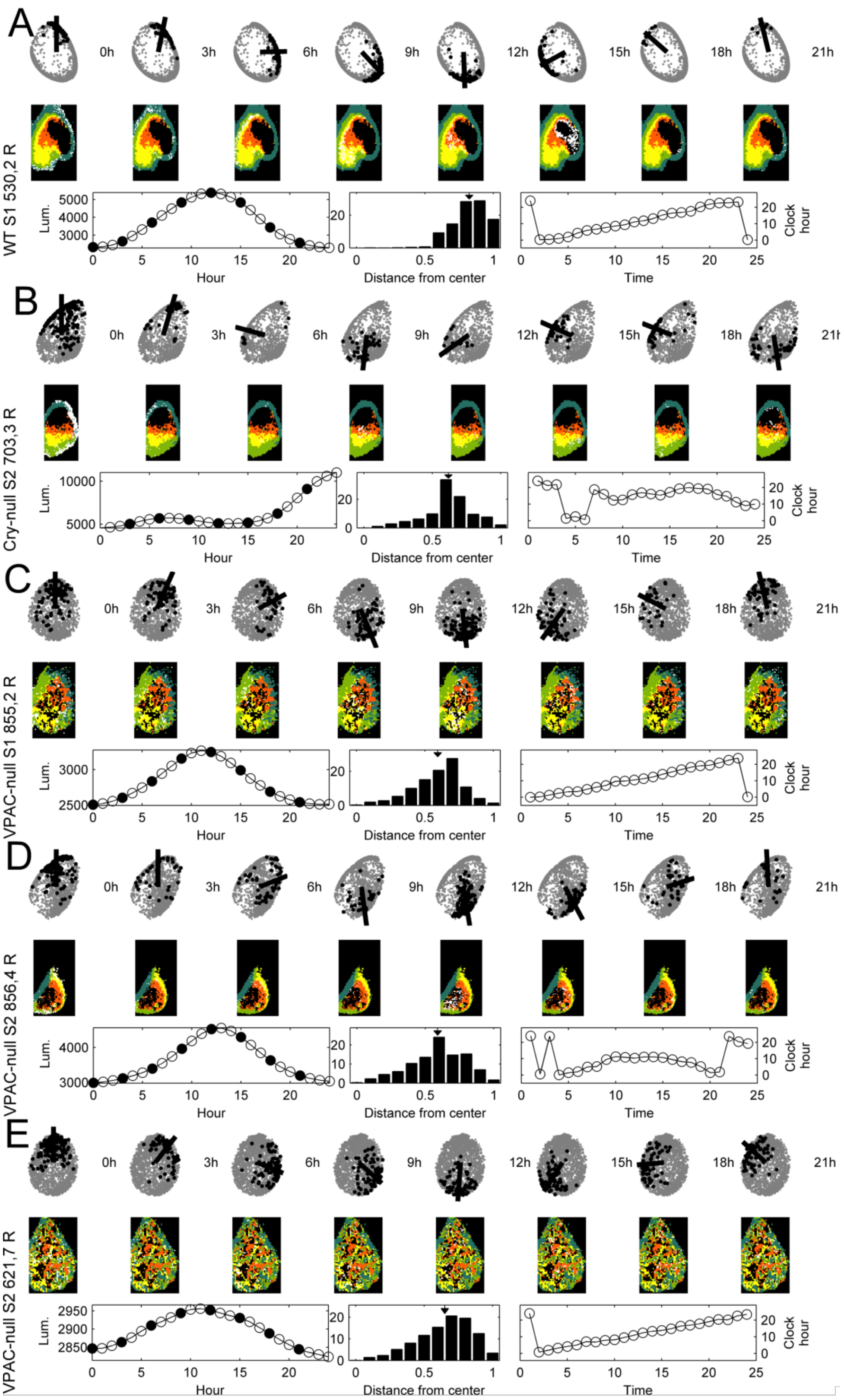

Figure S5: We show the same analyses as in Figure 6 for the right lobes of the SCN in that Figure.

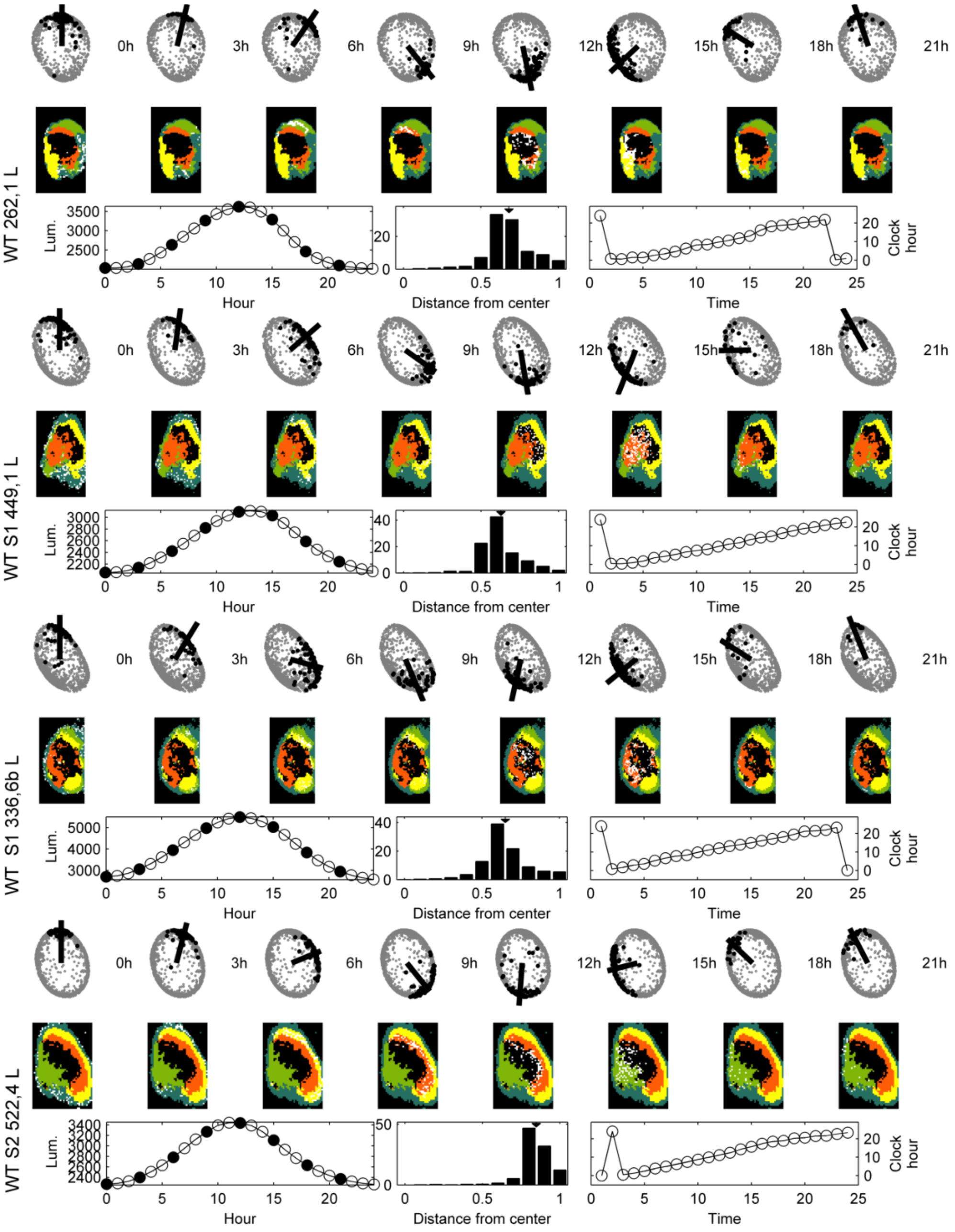

Figure S6: We show the results of following the procedure used to create Figure 6 for all additional WT animals (left lobes).

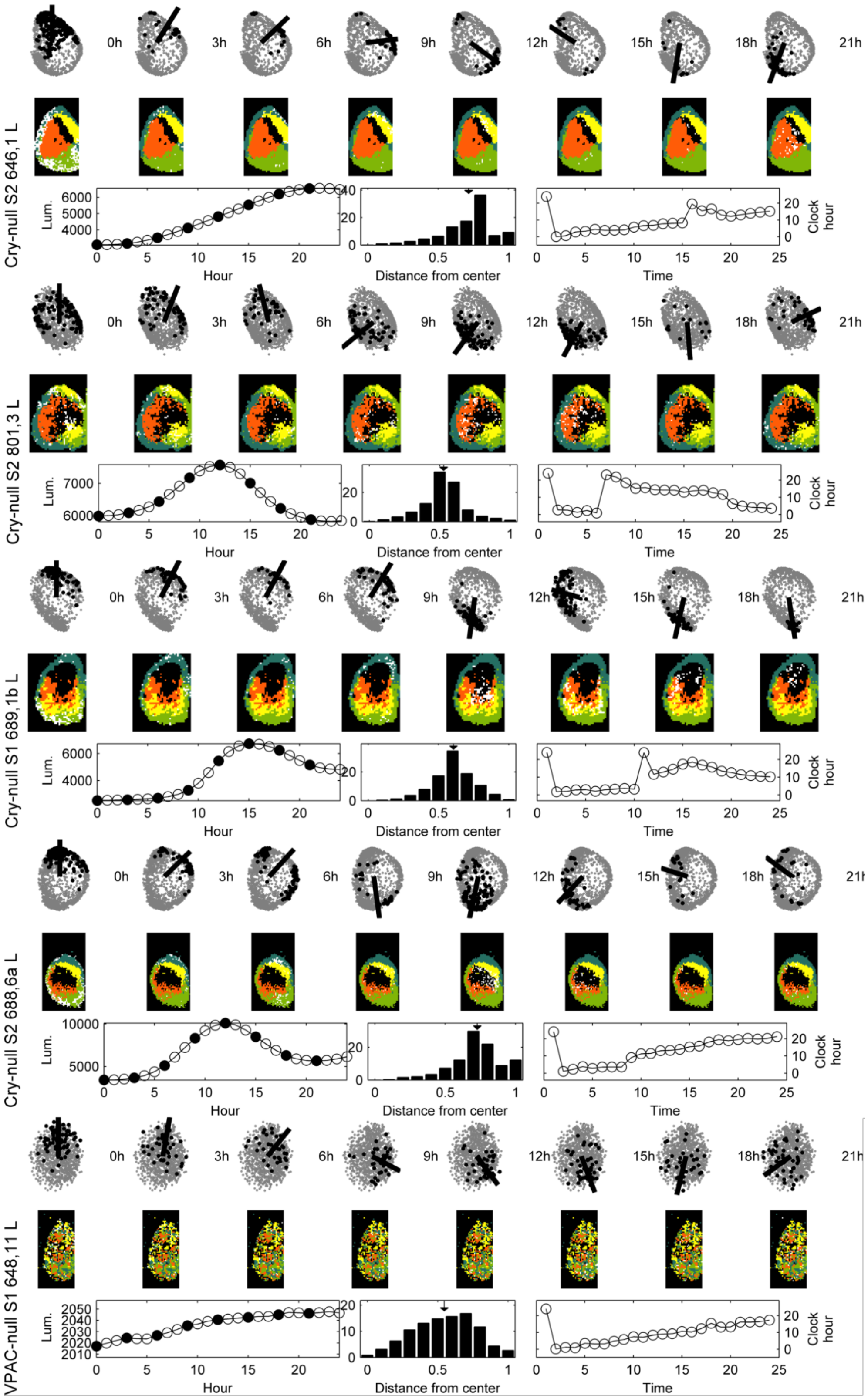

Figure S7: We show the results of following the procedure used to create Figure 6 for the left lobes of all additional Cry-null SCN (top rows) and one VPAC2-null SCN (last row).

Supplemental Movies: The spectral embedding movies show the link between spatial and temporal organization in a WT and CRY-null slice, depicting changes over the entire recording period. The images in the top row are taken from the same slice shown in Figure 6A. The "clock face" on the left side of the top row, shows the spectral embedding, where the white circles are associated with spatial locations with the highest bioluminescence. The "clock hand" gives the mean direction of the white circles. On the right side of the top row, the spatial locations in the SCN are also denoted by white pixels. In both images, the other locations are colored according to the clusters given by spectral clustering. In each case, the movie runs over the entire time series (in Figure 6 only 8 instances in time have been selected for display). The bottom row traces the progress through the mean time series for the SCN as the movie progresses.

SM1 corresponds to the slice in Figure 6A and SM2 corresponds to Figure 6B. The spectral embedding analysis supports the results of cluster analysis and further demonstrates the occurrence of repeated local temporal features: in particular there is a recurrent 'start' impetus that exists in both WT and Cry-null slices.

The similarity graph of an SCN

The spectral cluster analyses in this paper are based on a graph theoretic model of the SCN. The similarity graph represents the SCN as a graph consisting of a collection of nodes and edges between those nodes. For our application, we choose nodes to be the superpixels (described in the main text) and we place an edge between two nodes weighted according to the similarity of their temporal brightness profiles computed according some chosen function, such as correlation

or cosine distance. While we give the technical details below, the basic idea is that superpixels with similarly shaped time series will have connections with high weights between them while superpixels with significantly differently shaped time series will have low or zero weight connections.

Spectral Clustering Algorithm

This algorithm decomposes the tissue into regions with coherent behavior of luminescence time series subject to an important constraint, which is that spectral clustering produces a solution to the N-Cut problem, a graph decomposition optimization. The algorithm follows from an analysis of the eigendata of the graph Laplacian associated to a graph. The techniques here are standard and a complete discussion may be found in von Luxberg's tutorial (von Luxburg, 2007). Here we summarize briefly the variant of the technique used in our analyses. First, we generate an adjacency matrix, *A*, for a weighted undirected graph, as described in detail in the section below. Then we construct the graph Laplacian, $I - D^{-\frac{1}{2}}AD^{-\frac{1}{2}}$, where *I* is the identity matrix and $D_{ii} = \sum_j A_{ij}$ with all other entries being zero. This defines the so-called *symmetrized graph Laplacian* (note that there are several other definitions of the graph Laplacian). Second, we compute the eigenvalues and eigenvectors of *L*. It is well known (Chung, 1997) that the number of zero eigenvalues equals the number of connected components and, as the matrix is symmetric, *L* has a full complement of real eigenvalues. All of our graphs have one connected component and we order the eigenvalues from smallest to largest:

$$0 = \lambda_0 < \lambda_1 \leq \lambda_2 \leq \cdots \leq \lambda_n$$

The first nontrivial eigenvalue, λ_1, is called the Fiedler value (Fiedler, 1968) and describes the algebraic connectivity of the graph. To finish the implementation of spectral

clustering, we determine the number, *l*, of significant eigenvalues we wish to use. In the literature, this determination is often done using domain knowledge or estimation from a scree plot - we used the latter (see the main text for parameter estimation). Using the *l* associated eigenvectors, we construct the *spectral embedding* using the entries of eigenvectors associated to the first *l* nonzero eigenvalues as coordinates in $\mathbb{R}^l$. This embedding provides the geometric basis for spectral clustering – nodes are considered close to one another if they are close in this space, even if they are quite distant in other ways (e.g. spatially distant). Our last step in spectral clustering is to apply *k*-means to the spectral embedding to cluster the nodes together. To use spectral clustering we must specify three parameters, the scale parameter σ (described below), the number of eigenvectors *l*, and the number of clusters *k*.

Spectral embedding: Similarity model of the SCN

The spectral embedding is a mapping of the superpixels to the plane which allows us to see the extent to which the temporal and spatial structures of PER2 concentrations in the SCN are linked. Key to encoding the link between temporal and spatial structures in the spectral embedding is choosing a good adjacency matrix for our graph model of the functional connectivity SCN. To specify our adjacency matrix, we need to specify a notion of dissimilarity between the superpixels. Our notion is built from correlation between the time series of luminescence profiles for the superpixels. Precisely, we first normalize our time series by removing the mean time series via projection. If T_i is the time series associated with superpixel i, we let

$$\hat{T}_i = T_i - proj_{T_m} T_i = T_i - \frac{T_i \cdot T_m}{|T_m|^2} T_m,$$

where T_m is the mean time series. We then construct the correlation matrix of the normalized time series - $C_{ij} = corr\ (\hat{T}_i, \hat{T}_j)$. We convert the correlation to half the distance on the sphere by letting $d_{ij} = \sin\left(\frac{\cos^{-1} C_{ij}}{2}\right)$. In other words, if we further ensure that the normalized time series are of unit length, then d_{ij} is half the chordal distance on the unit sphere.

This distance matrix is a dissimilarity matrix, while an adjacency matrix for the graph needs to be a similarity matrix. We nonlinearly rescale the distance matrix to make a similarity matrix suitable for use as an adjacency matrix:

$$A_{ij} = e^{-\frac{d_{ij}^2}{\sigma^2}}.$$

The parameter σ is a scale parameter – it allows us to pick which distances (and hence correlations) we consider to be relevant and which are to be devalued (see below for parameter estimation).

Spectral clustering and graph cut problems:

To motivate our use of spectral clustering, we briefly review a connection to graph partitioning. One of the fundamental problems in graph theory is the cut problem. In the cut problem, we wish to partition the graph into two (or more) pieces while doing the least total damage to the network. To make this precise, if the nodes of the graph are divided between two sets, B and B^C, the cut of the graph is defined to be $cut(B, B^C) = \sum_{i \in B, j \in B^C} A_{ij}$ where A_{ij} is the weight of the edge connecting node *i* and node *j*. Thus, the cut problem is solved by finding the set B which minimizes $cut(B, B^C)$. While this formulation is intuitive, it has some problems for specific types of graphs. For example, if the graph has a node with a single edge, it is often the optimal

solution to the cut problem to let that node be the set B. Often, this is not the "correct" solution in the sense that it does not meet the criteria of the practitioner. In particular, it is often desirable to have a partition which divides the graph into roughly equal pieces. In this respect, the cut problem often fails. To mitigate this, we can introduce the normalized cut problem (see (Shi & Malik, 2000). In this problem, we minimize the normalized cut:

$$NCut(B,B^C) = \frac{cut(B,B^C)}{\sum_{i\in B,j} A_{ij}} + \frac{cut(B,B^C)}{\sum_{i\in B^C,j} A_{ij}}$$

Minimizing this generally forces the two sets, B and B^C to be of roughly equal size. Both these problems, as well as other variants, are NP-hard problems. But, relaxations of these problems turn out to be simply linear algebra problems. In fact, the solution to the relaxed normalized cut problem (for a single cut) is given by the Fiedler vector of the graph Laplacian we described above (the set B is defined by the nodes which are associated with positive entries in the Fiedler vector). The solution to the relaxed problem for a partition into *k* subsets is given by spectral clustering.
